# Design, Synthesis, Structure and Properties of Ba-Doped Derivatives of SrCo_0.95_Ru_0.05_O_3−δ_ Perovskite as Cathode Materials for SOFCs

**DOI:** 10.3390/ma12121957

**Published:** 2019-06-18

**Authors:** Sabina Sydyknazar, Vanessa Cascos, Loreto Troncoso, Ana Laura Larralde, María Teresa Fernández-Díaz, José Antonio Alonso

**Affiliations:** 1 Instituto de Ciencia de Materiales de Madrid, C.S.I.C., Cantoblanco, E-28049 Madrid, Spain; sabisha.es@gmail.com (S.S.); vcascos@icmm.csic.es (V.C.); ja.alonso@icmm.csic.es (J.A.A.); 2 Instituto de Materiales y Procesos Termomecánicos, Universidad Austral de Chile, General Lagos, 2086, 5111187 Valdivia, Chile; 3 INQUIMAE, Departamento de Química Inorgánica, Analítica y Química Física, Facultad de Ciencias Exactas y Naturales, Universidad de Buenos Aires, Buenos Aires C1428EGA, Argentina; alarralde@qi.fcen.uba.ar; 4 Institut Laue Langevin, BP 156X, F-38042 Grenoble, France; ferndiaz@ill.eu

**Keywords:** IT-SOFC, cathode, perovskite, neutron diffraction, SrCoO_3_

## Abstract

We have designed and prepared a novel cathode material for solid oxide fuel cell (SOFC) based on SrCo_0.95_Ru_0.05_O_3−δ_ perovskite. We have partially replaced Sr by Ba in Sr_0.9_Ba_0.1_Co_0.95_Ru_0.05_O_3−δ_ (SBCRO) in order to expand the unit-cell size, thereby improving the ionic diffusion of O^2−^ through the crystal lattice. The characterization of this new oxide has been studied at room temperature by X-ray diffraction (XRD) and neutron powder diffraction (NPD) experiments. At room temperature, SBCRO perovskite crystallizes in the P4/mmm tetragonal space group, as observed from NDP data. The maximum conductivity value of 18.6 S cm^−1^ is observed at 850 °C. Polarization resistance measurements on LSGM electrolyte demonstrate an improvement in conductivity with respect to the parent Sr-only perovskite cathode. A good chemical compatibility and an adequate thermal expansion coefficient make this oxide auspicious for using it as a cathode in SOFC.

## 1. Introduction

The implementation of the hydrogen economy requires the combination of renewable energies used for the production of hydrogen, and the utilization of this gas in fuel cells to recover the electrical energy and water as a byproduct. Fuel cells are called to replace combustion engines, showing great potential as energy conversion devices; they are able to transform the chemical energy of hydrogen directly into electrical energy with high efficiency and cleanness [1,2,3]. Among the fuel cells, the Solid Oxide Fuel Cells (SOFC) involve the most demanding technology from the point of view of the material selection. Due to the high operating temperatures (800–1000 °C), the enhanced reaction kinetics translates high efficiency in energy conversion, eliminating the problems that arise from the use of liquid electrolytes. As a drawback, these high operating temperatures mean a demanding stress for the different components, electrodes, solid electrolytes and interconnectors. Therefore, a reduction of the working temperature is mandatory, which entails the adoption of more performant materials to avoid diminishing the oxygen-reduction kinetics at the air electrode (cathode). An important strategy is to increase the active area of the electrode-electrolyte interface, with the development of new electrode materials that are able to transport ions and electrons simultaneously (known as Mixed Ionic and Electronic Conductors, MIECs) [4]. The use of MIEC materials reduces mechanical and chemical compatibility issues. Most of the known MIEC materials belong to the ABO_3_ perovskite family, where A and B can be partially or totally substituted leading to a wide range of catalytic activities, oxygen stoichiometries and interesting transport properties [5,6].

The high-temperature cubic phase of SrCoO_3−δ_ is a promising material due to its high electrical conductivity and oxygen permeation flux [7,8]. Depending on the synthesis conditions and the oxygen content, we can obtain different structural distortions such as orthorhombic, tetragonal, and cubic unit cells. Whereas the cubic phase exhibits an excellent ionic and electronic conductivity, the hexagonal phase has very poor properties and, unfortunately, it is thermodynamically stable, which limits the use of this pure material as a cathode in SOFC. As a strategy to prevent this cubic-to-hexagonal phase transition, it was found that replacing few percentages of Co by aliovalent elements like Re, Nb, Sb or Ti was effective to stabilize the cubic phase in a wide temperature range [9,10,11,12,13]. Recently, we observed that replacing 5% of Co by Ru prevents this unwanted transformation and enhances the catalytic oxygen reduction reaction [14]. In present research, we worked on the parent SrCo_0.95_Ru_0.05_O_3−δ_ with an additional Ba doping at the Sr positions, in order to expand the unit-cell volume, so as to enhance the ion diffusion of O^2−^ ions through the crystal lattice in this MIEC oxide. Our goal was to improve the electrochemical, electrical and catalytic performance of this compound in order to use it as a cathode in a SOFC.

This work describes the full structural characterization of the novel material, including neutron powder diffraction data, in complement with transport, dilatometric, and polarization resistance measurements that confirm the excellent performance of this material as cathode in SOFCs.

## 2. Experimental

### 2.1. Synthesis

Sr_0.9_Ba_0.1_Co_0.95_Ru_0.05_O_3−δ_ perovskite was obtained as a polycrystalline powder via soft chemistry, using the citrate-nitrate route. Stoichiometric amounts of Sr(NO_3_)_2_ (Strem Chemicals, Newburyport, MA, USA, 99%), Ba(NO_3_)_2_ (Merck, Kenilworth, NJ, USA, 99%) and Co(NO_3_)_2_∙6H_2_O (Alfa Aesar, Haverhill, MA, USA, 99%) were dissolved in a 10% saturated solution of citric acid. Afterwards, RuO_2_ (Alfa Aesar, 99.9%) was added as a powder and kept in suspension until the solution was slowly evaporated and an organic resin was formed. The latter was dried at 120 °C and decomposed at 600 °C for 12 h. Finally, the solid was fired in air at 1000 °C for 24 h and used without further treatment.

### 2.2. Structural Characterization

The product was characterized by X-ray diffraction (XRD) for phase identification and to assess phase purity. The measurement was performed using a Bruker-AXS D8 diffractometer (Bruker, Billerica, MA, USA, 40 kV, 30 mA) in Bragg-Brentano reflection geometry with CuK_α_ radiation (λ = 1.5418 Å), in the 10–64° 2θ range. A nickel filter allowed the elimination of Cu K_β_ radiation.

In order to characterize the oxygen vacancy distribution, a neutron powder diffraction (NPD) study was carried out. A high-resolution pattern was collected in the D2B diffractometer at the Laue-Langevin Institute (Grenoble, France) with a neutron wavelength λ = 1.594 Å within the angular range of 2θ from 10 to 160°. About 2 g of the sample was contained in a cylindrical vanadium holder. A time of 2 h was required to collect a full diffraction pattern at 25 °C. The diffraction data were analyzed by the Rietveld method through the FULLPROF program (version 3.00) [15]. A pseudo-Voigt function was chosen to generate the line shape of the diffraction peaks. The scale factor, background points, zero shift, half-width, pseudo-Voigt corrected for asymmetry parameters, unit-cell parameters, positional coordinates and isotropic displacement factors for metals and anisotropic for oxygen atoms were refined in the final run. Occupancy factors for oxygen atoms were also refined from the collected NPD data.

### 2.3. Thermal Expansion Measurements and Chemical Compatibility

The mechanical compatibility between this material with the other cell components was determined by thermal expansion measurements in a cylindrical dense pellet (5 mm diameter × 2 mm thickness) obtained by pressing the prepared powder under 200 MPa of uniaxial pressure and sintered at 1050 °C for 12 h in air. The thermal expansion of the sintered sample was studied using a LINSEIS L75/155 °C dilatometer (Linseis Messgeraete, Selb, Germany) between 400 and 900 °C in air.

### 2.4. dc Measurements

The electrical conductivity was measured in the temperature range between 20 and 850 °C, using the *dc* four-probe technique in bar-shaped pellets under *dc* currents between 0.01 and 0.5 A. The currents were applied and collected using a Potentiostat-Galvanostat AUTOLAB PGSTAT 302 from ECO CHEMIE (NorECs Norwegian Electro Ceramics AS, Oslo, Norway).

### 2.5. ac Measurements

Electrochemical impedance spectroscopy (EIS) measurements were performed in air under open-circuit potential (OCP) conditions in a symmetrical configuration to extract the corresponding values of the electrolyte and electrode contributions. A dense electrolyte pellet of LSGM (La_0.83_Sr_0.17_Ga_0.8_Mg_0.2_O_3−δ_) was used and was prepared in our laboratory. An ink of the cathode material was created with the Sr_0.9_Ba_0.1_Co_0.95_Ru_0.05_O_3−δ_ powders. First, the material was ball-milled in ethanol to break the agglomerates and then the dried powders were mixed with a binder (V-006 from Heraeus, Madrid, Spain). The ink was symmetrically painted over the electrolyte onto both surfaces in Sr_0.9_Ba_0.1_Co_0.95_Ru_0.05_O_3−δ_/LSGM/Sr_0.9_Ba_0.1_Co_0.95_Ru_0.05_O_3−δ_ configuration. The cells were calcined at 1000 °C for 2 h to obtain a good adherence between the cathodes and the electrolytes according to a procedure previously optimized in our laboratories for doped-SrCoO_3_ cathode powders. Subsequently, two Pt electrodes were painted onto the cathode surfaces and calcined at 800 °C for 1 h to ensure equipotential conditions. EIS was then performed in potentiostatic mode, decreasing the temperature from 900 to 600 °C, with an excitation voltage of 50 mV in the range of 1 kHz to 100 MHz. All the cell impedances were normalized by the superficial area so that they have the units of Ω cm^2^. The electrode endurance was evaluated under a constant cathodic current density of 200 mA·cm^−2^ at 850 and 800 °C for 20 h. The current passage was interrupted to collect the electrochemical impedance spectra in open circuit. The data obtained were analyzed with the Zview software (version 3.5) (Scribner Associates). The EIS spectra were collected using an AUTOLAB PGSTAT 302 (NorECs Norwegian Electro Ceramics AS, Oslo, Norway) from ECO CHEMIE.

## 3. Results and Discussion

### 3.1. Crystallographic Characterization

**Figure 1:**
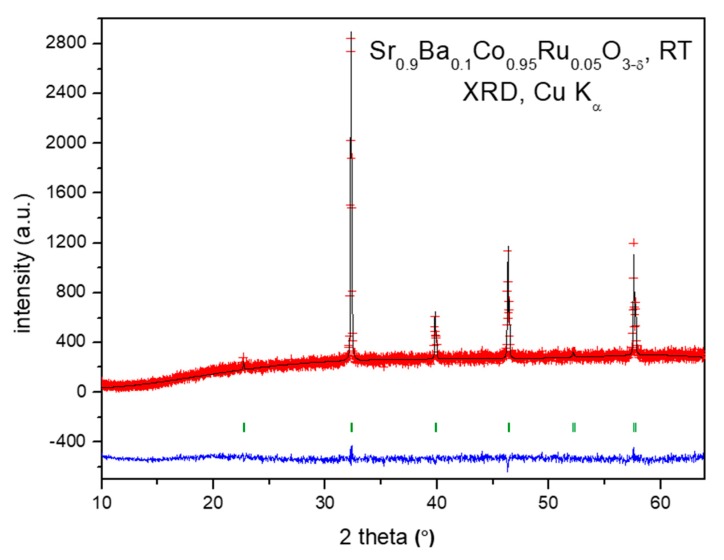
Observed (red crosses) and calculated profiles (full black line) for Sr_0.9_Ba_0.1_Co_0.95_Ru_0.05_O_3−δ_ perovskite after Rietveld refinement of the crystal structure in a standard perovskite model in the Pm-3m space group, with unit-cell parameter of *a* = 3.9159(5) Å. The difference plot (full blue line) and allowed Bragg reflections (green vertical lines) are also shown.

Sr_0.9_Ba_0.1_Co_0.95_Ru_0.05_O_3−δ_ perovskite was obtained as a polycrystalline powder. The XRD diffractogram (Figure 1) collected with Cu Kα radiation evidenced the presence of the perovskite cubic phase. In a first approach, the crystal structure was refined on the basis of a simple cubic aristotype defined in the standard perovskite model at Pm-3m space group, with unit-cell parameter of *a* = 3.9159(5) Å. This is considerably expanded with respect to that corresponding to the parent compound of 3.858(1) Å [14], as expected for the much bigger size of Ba^2+^ (1.61 Å) with respect to Sr^2+^ (1.44 Å) ions in twelvefold coordination [16].

**Figure 2:**
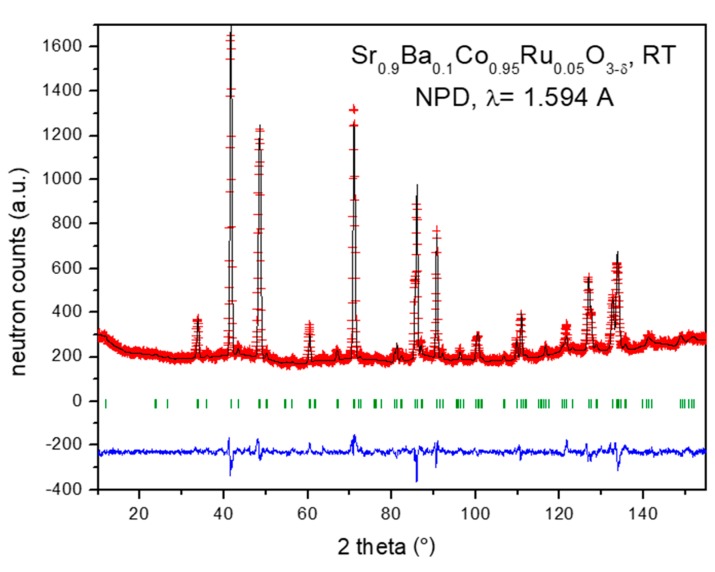
Observed (crosses), calculated (full line) and difference (at the bottom) neutron powder diffraction (NPD) profile for Sr_0.9_Ba_0.1_Co_0.95_Ru_0.05_O_3−δ_ phase at 25 °C, refined in the tetragonal P4/mmm space group. The vertical markers correspond to the allowed Bragg reflections.

A room temperature study of Sr_0.9_Ba_0.1_Co_0.95_Ru_0.05_O_3−δ_ by neutron powder diffraction (NPD) was very useful to investigate fine structural details. Although the XRD pattern can be interpreted in a cubic model, the NPD data suggest the presence of a tetragonal superstructure with a doubled *c*-axis, as *a* = *b* ≈ *a*_0_, *c* ≈ 2*a*_0_ and it was correctly refined in the P4/mmm space group. In this perovskite, the Sr and Ba atoms are distributed at random at *2h* (½, ½, z) sites, Co1 at *1a* (0, 0, 0), Co2 and Ru at *1b* (0, 0, ½), and the three types of oxygen atoms O1 at *2f* (½, 0, 0), O2 at *2g* (0, 0, z) and O3 at 2e (½, 0, ½) sites. The occupancy factors of the three types of oxygen atoms were refined. While O2 and O3 displayed conspicuous deficiency, O1 converged to values close to the stoichiometric value, so the occupancy factor was set to unity. Ru was unambiguously located at Co2 sites, thanks to the contrast between scattering lengths for Co (2.49 fm) *vs* Ru (7.03 fm). Figure 2 illustrates the goodness of the fit for the NPD pattern for the Sr_0.9_Ba_0.1_Co_0.95_Ru_0.05_O_3−δ_ compound. Table 1 shows the final structural parameters, the displacement factors and the agreement factors after the Rietveld refinement from NPD data at room temperature.

**Figure 3:**
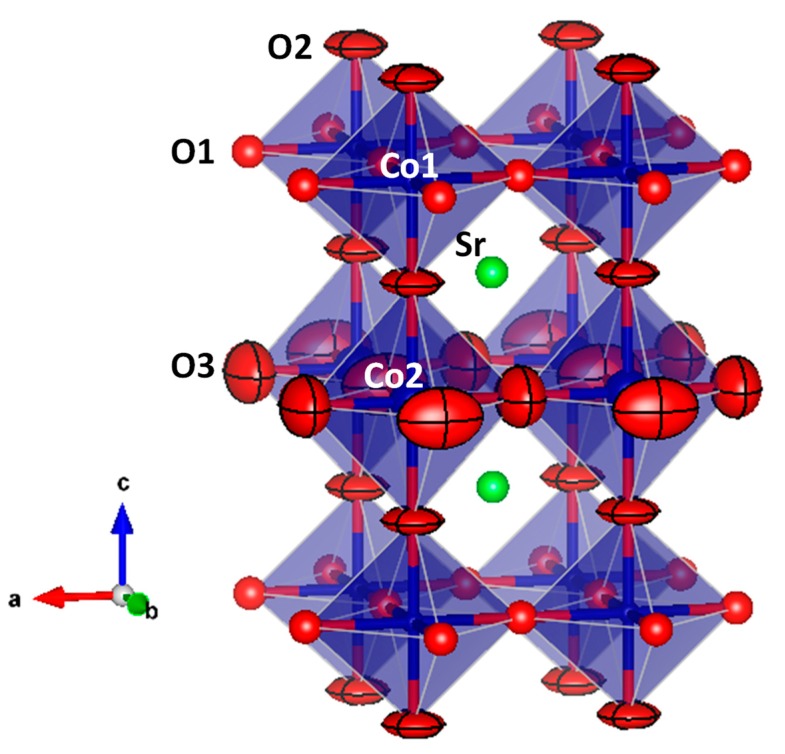
Tetragonal crystal structure observed for Sr_0.9_Ba_0.1_Co_0.95_Ru_0.05_O_3−δ_.

The tetragonal crystal structure (Figure 3) can be defined as a superstructure of perovskite with a doubled *a_0_* axis along the *c* direction. This superstructure is due to the long-range ordering of the oxygen vacancies, quantified in the neutron refinement, and concentrated at the O2 and O3 positions. For this reason, the superstructure could not be detected from XRD data, given the weak scattering factor for oxygen atoms. The *z*(O2) coordinate (Table 1) is slightly shifted from ¾, determining alternating elongated (Co,Ru)_2_O_6_ octahedral layers, containing Co2 atoms, and flattened Co1O_6_ octahedral layers containing Co1 atoms. The (Co,Ru)_2_O_6_ octahedra share O3 atoms, while Co1O_6_ octahedra share O1 atoms. Finally, both octahedra layers share O2 atoms. These differences in the octahedral size suggest that Co1 cations might present a higher oxidation state, closer to 4+, whereas Co2 is close to 3+, assuming a full charge disproportionation. In fact, the presence of Co^3+^ with an intermediate-spin state in Co2 sites could cause the Jahn-Teller distortion of the Co–O bond lengths, and O^2−^ ions would approach Co^4+^ cations, moving from Co2 to Co1, justifying the observed shift of O2 atoms.

### 3.2. Thermal Expansion Measurements

**Figure 4:**
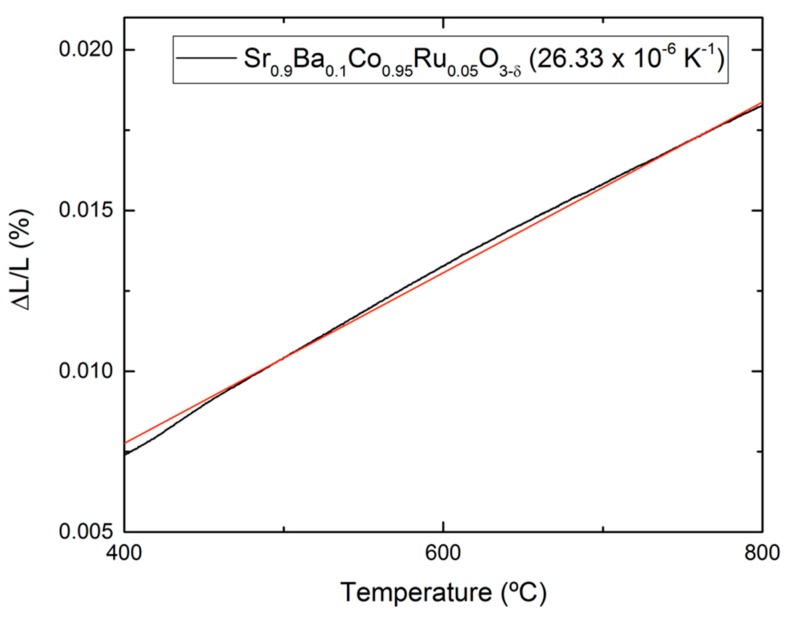
Thermal expansion determined by dilatometry of the perovskite Sr_0.9_Ba_0.1_Co_0.95_Ru_0.05_O_3−δ_ collected in two consecutive heating runs.

The thermal expansion coefficient (TEC) was measured by dilatometric analysis, in order to determine the mechanical compatibility of the material with the components of the cell. The analysis was carried out between 400 and 900 °C for several cycles and the data were collected only during the heating runs. Figure 4 shows the thermal expansion of Sr_0.9_Ba_0.1_Co_0.95_Ru_0.05_O_3−δ_, which is approximately linear in the considered temperature range, and does not display discontinuities that could suggest decompositions. The TEC parameter, measured in air between 400 and 850 °C, is also shown. The determined value of TEC for the prepared oxide is substantially higher than those that normally have the components of the cell, but it is typical for cobaltites, which have been successfully used as cathodes in SOFC. In fact, the porous nature of the electrode once it is deposited on the electrolyte is able to accommodate the excessive dilation upon heating, avoiding cracking problems or delamination from the electrolyte.

### 3.3. Chemical Compatibility

**Figure 5:**
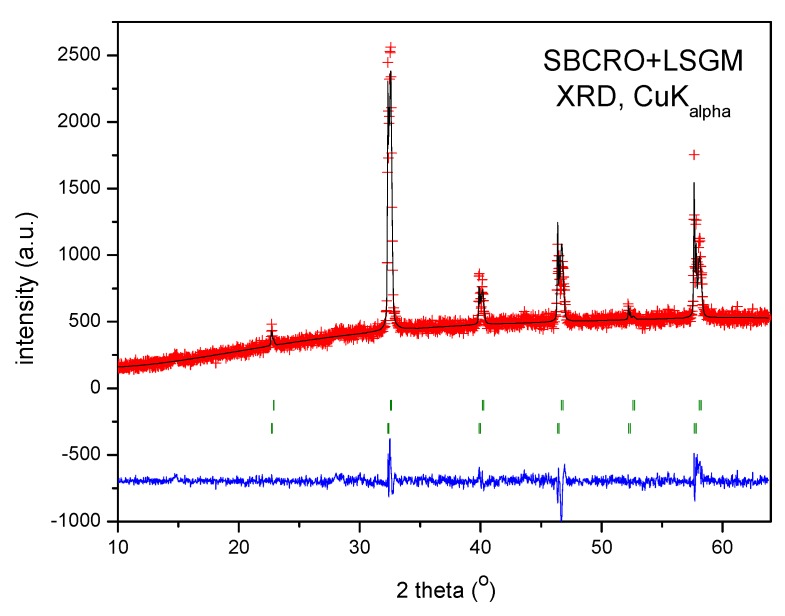
Rietveld refinement from XRD data of a powdered mixture of the La_0.83_Sr_0.17_Ga_0.8_Mg_0.2_O_3−δ_ (LSGM) electrolyte and the Sr_0.9_Ba_0.1_Co_0.95_Ru_0.05_O_3−δ_ electrode after a thermal treatment in air at 1100 °C, showing both phases unchanged.

The chemical compatibility of the Sr_0.9_Ba_0.1_Co_0.95_Ru_0.05_O_3−δ_ sample with the La_0.83_Sr_0.17_Ga_0.8_Mg_0.2_O_3−δ_ (LSGM) electrolyte has been studied by mixing and grinding both powdered materials in the same quantities and heating the mixture at 1100 °C in air for 12 h. Figure 5 shows a Rietveld analysis of the product, consisting of un unaltered mixture of the starting Sr_0.9_Ba_0.1_Co_0.95_Ru_0.05_O_3−δ_ and LSGM oxides. Both are cubic perovskites with slightly different unit-cell parameters. No reaction between the materials was observed; tiny peaks at 2θ = 15°, 28° and 44° were attributed to a minor impurity phase below the 0.5% level.

### 3.4. Electrical Conductivity Measurements

#### 3.4.1. *dc* Measurements

**Figure 6:**
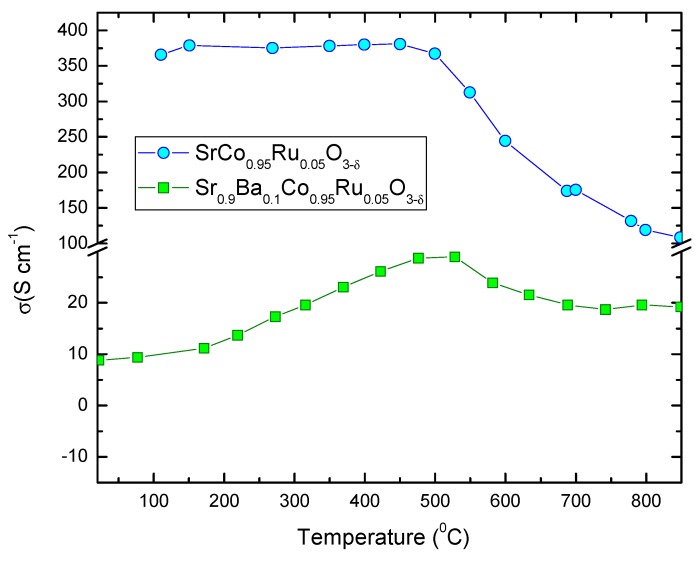
Thermal evolution of the *dc* electrical conductivity of Sr_0.9_Ba_0.1_Co_0.95_Ru_0.05_O_3−δ_ compared with the parent compound Sr_0.9_Co_0.95_Ru_0.05_O_3−δ_.

Figure 6 shows the thermal variation of the electrical conductivity of the Sr_0.9_Ba_0.1_Co_0.95_Ru_0.05_O_3−δ_ perovskite, measured on a sintered bar (10 mm × 3 mm × 3 mm) in air by the four-probe technique. It is compared with that of the parent compound, SrCo_0.95_Ru_0.05_O_3−δ_. In the low-temperature region, it shows semiconductor behavior with an anomaly at 450 °C, suggesting an insulator-to-metal transition. Above this temperature, a negative slope is observed until 720 °C. The maximum conductivity value at 450 °C is 28.5 S cm^−1^; the conductivity at 850 °C is 18.6 S cm^−1^. Although the electrical conductivity is lower than those presented by other Co-perovskites, with values between 30 and 50 S cm^−1^, it should be sufficient for this material to be used satisfactorily as cathode material. The anomaly at 450 °C is also present in the parent compound (Figure 6, upper curve, taken from [14]), where it was related to a structural rearrangement from the room-temperature tetragonal superstructure to a truly simple cubic structure, where all the oxygen atoms become equivalent [14]. It is worth noting that the incorporation of Ba^2+^ ions to the perovskite structure, expanding the unit-cell size, separate apart the Co–O atoms decreasing the orbital overlap, and for that reason the *dc* conductivity values decrease with respect to the value observed for the parent compound at 850 °C of 110 S cm^−1^.

#### 3.4.2. *ac* Measurements

Electrochemical impedance spectroscopy (EIS) determinations seek to separate and identify the global electrode reactions into different contributions evidenced by distinct impedance arcs. The reaction steps are time-dependent and are represented in terms of frequency (s^−1^). Each charge-transfer limiting process (i.e., electrolyte, electrode process, etc.) occurs at a distinct characteristic frequency, resulting in a separate arc of the impedance diagram [15,16]. The time-response of each individual process for the oxygen reduction reaction (ORR) is often modeled in terms of an equivalent circuit. The high frequency (HF) contribution is attributed to the oxygen-ion transfer process from the electrode to the electrolyte. A medium frequency (MF) signal is assigned to oxygen ion diffusion co-limited by charge transfer, and a low frequency (LF) region due to dissociated absorption [17].

The polarization resistance was investigated by *ac* impedance spectroscopy in the temperature range of 600 to 900 °C. The Pt/Sr_0.9_Ba_0.1_Co_0.95_Ru_0.05_O_3−δ_/LSGM/Sr_0.9_Ba_0.1_Co_0.95_Ru_0.05_O_3−δ_/Pt symmetrical cell was previously sintered at 1000 °C for 2 h. The data, collected in air, were normalized for the geometric area of the electrodes in the symmetrical cell and halved due to the symmetry of the cell.

The electrochemical characterization shows the same behavior in all the temperature range: (1) an inductive process, product of the electrochemical equipment and Pt wires, (2) an ohmic contribution, i.e., the intersection of the spectrum with the x-axis due to the internal resistance of the LSGM electrolyte, and (3) two semicircles, representing the polarization resistance of the cathode. These last features suggest the presence of two internal processes occurring at the triple phase boundary (TPB): the first one, at high frequencies, corresponds to the electrode/electrolyte interfacial charge transfer resistance, and the second one, at lower frequencies, associated with the oxygen reduction reaction (ORR).

**Figure 7:**
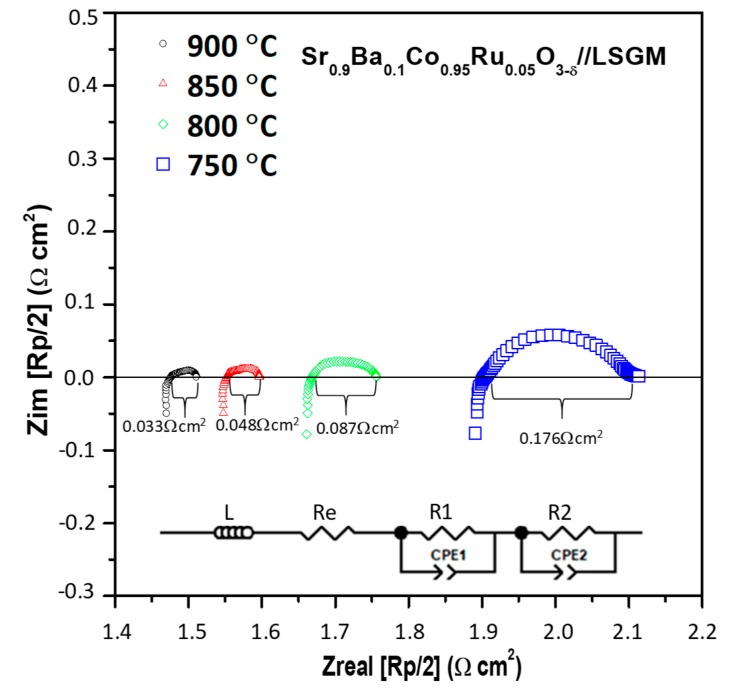
Impedance spectra obtained at different temperatures in symmetrical cells of Sr_0.9_Ba_0.1_Co_0.95_Ru_0.05_O_3−δ_ onto LSGM electrolyte.

Figure 7 displays the impedance diagrams of the cathode in the temperature range between 750 and 900 °C, showing that the resistance decreases as the temperature increases, as expected. All the spectra were fitted with the equivalent circuit indicated in the inset, using the non-linear least squares fitting of the Z-view program. In the scheme, L represents the inductive process, Re the electrolyte resistance, R1 the charge transfer resistance and R2 the ORR resistance. As mentioned, the sum of R1 and R2 corresponds to the polarization resistance Rp. The fact that the electrode can be polarized by the charge transfer and reduction reactions, implies the appearance of capacitive processes (“double layer” effect), which occurs in parallel to the resistive processes. This is represented in the circuit as a constant phase element (CPE), which represents a time-dependent capacitive element (pseudo-capacitance). The obtained Rp values are presented in Table 2. For comparative reasons, reported Rp values (studied previously by our group) for several SrCo_1−x_M_x_O_3_ (M = Ru, Re, Ti, V) perovskites are also included. They were all studied following the same guidelines in terms of preparation and sintering processes.

Rp values obtained for Sr_0.9_Ba_0.1_Co_0.95_Ru_0.05_O_3−δ_ are comparable to those previously reported. From the data in Table 2, we can see that not necessarily only a lower polarization resistance will imply a higher power density in a single test cell. In the case of the V^5+^ doped sample, the Rp is only 0.025 Ω cm^2^, and its power density is 550 mW cm^−2^; instead, for the 10% Ru^4+^ doped sample, the Rp is 0.110 Ω cm^2^ and its power density is 652 mW cm^−2^, both at 850 °C. This suggests that the doping cation must also have a functional catalytic activity for the ORR reaction. Comparing the results gathered in Table 2, it can be clearly seen that the one that outperforms under these conditions is the Ru^4+^ cation with 5% of doping. The value of 0.048 Ω cm^2^ determined for the present Sr_0.9_Ba_0.1_Co_0.95_Ru_0.05_O_3−δ_ sample (vs. 0.070 Ω cm^2^ for the parent compound) brings us to the conclusion that the introduction of Ba^2+^ into the Sr site effectively decreases the polarization resistance, probably as an effect of the oxide-ion diffusion across the crystal lattice. This is auspicious regarding the performance of this material as cathode for SOFC in the intermediate temperature range.

**Figure 8:**
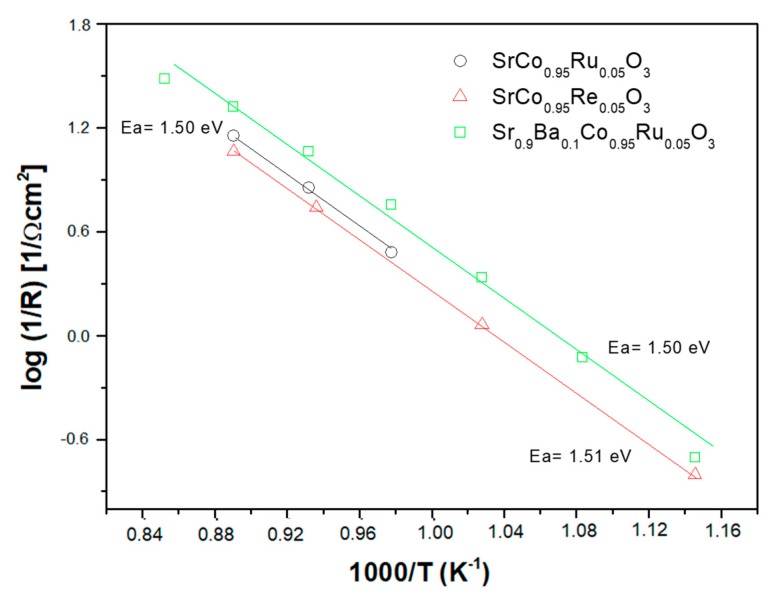
Arrhenius plot for Sr_0.9_Ba_0.1_Co_0.95_Ru_0.05_O_3_ compound compared with similar cathodes.

The activation energy (*E_a_*) for Sr_0.9_Ba_0.1_Co_0.95_Ru_0.05_O_3−δ_ can be obtained from the Arrhenius plots of the resistance as a function of temperature (Figure 8). This value of activation energy, *E_a_* = 1.50 eV, is associated with the process of oxygen reduction reaction and is comparable to that observed for electrodes of the SrCo_1−x_M_x_O_3−δ_ family, also included in Figure 8. It is also noticeable that the presented Ba-doped electrode presents an improvement in the total conductivity with respect to the parent Ru-containing compound.

**Figure 9:**
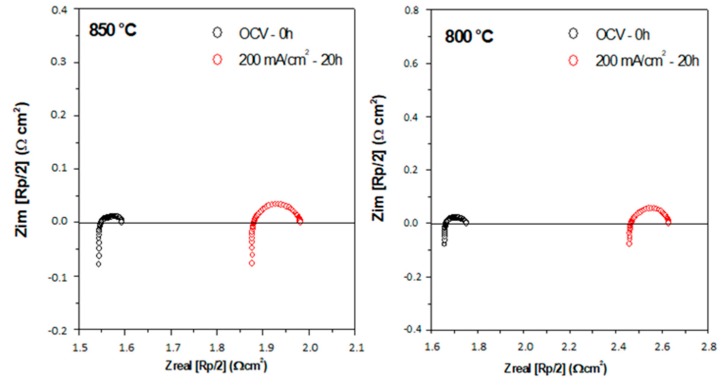
Impedance spectra of symmetrical cells under open circuit voltage (OCV) (0 h) and constant current density of 200 mA cm^−2^ (20 h) at (**a**) 850 °C and (**b**) 800 °C.

The electrode endurance was studied in open circuit conditions and *dc* current polarization. Figure 9 shows the symmetric cell impedance under OCV at 0 h compared with a constant current density of 200 mA cm^−2^ after 20 h and 40 h at 850 °C and 800 °C. It is possible to observe that under the electrical flow of direct current, the *dc* current flux produces an alteration in the electrode polarization resistance, increasing the electrolyte resistance and the polarization resistance, probably indicating an incipient reaction in the interface.

### 3.5. Scanning Electron Microscopy

**Figure 10:**
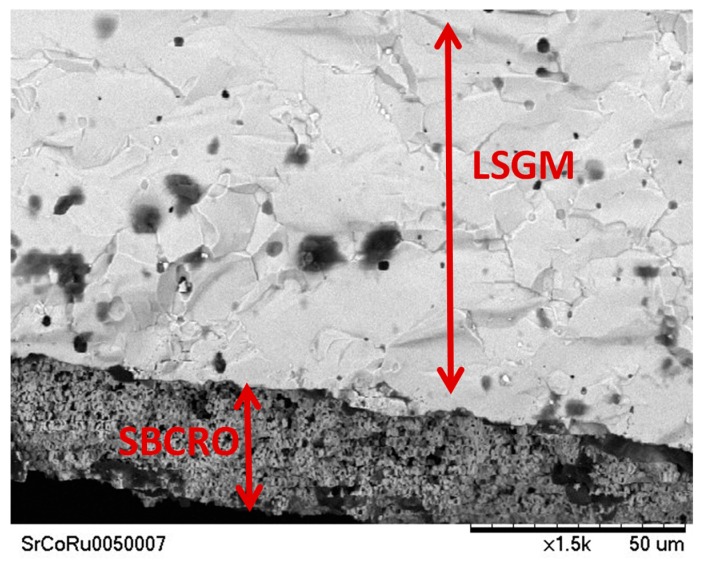
SEM micrograph showing the porous cathode layer of Sr_0.9_Ba_0.1_Co_0.90_Ru_0.10_O_3−δ_ tightly adhered to the dense LSGM electrolyte. The thickness of the cathode is about 25 μm.

Figure 10 shows a SEM post-mortem study of the pellet utilized in the EIS tests. The micrograph shows the interphase of the Sr_0.9_Ba_0.1_Co_0.95_Ru_0.05_O_3−δ_ cathode and the electrolyte (LSGM).

An important requirement for the most favorable cathode materials is the porosity. Figure 10 shows a good porosity in the surface of the cathode that favors the diffusion and reduction process of the oxygen throughout the bulk of the cathode. Also, the dense electrolyte layer without cracks or fractures is displayed.

## 4. Conclusions

In the present work, we have designed a novel cathode material by partially replacing Sr by Ba in SrCo_0.95_Ru_0.05_O_3−δ_ perovskite. We have shown with an NPD study that this compound is stabilized in a tetragonal perovskite structure at RT, defined in the P4/mmm group, with an expanded unit-cell volume with respect to the parent compound. The tetragonal superstructure is due to the long-range ordering of oxygen vacancies along the *c*-axis. The *dc* conductivity seems to be sufficient for a good performance as cathode material in SOFC. A good chemical compatibility was observed with the LSGM electrolyte for 12 h. The TEC of this material is higher than those usually presented in the other cell components, but it is characteristic of perovskites that contain Co. Interestingly, an EIS investigation unveils a lower polarization resistance than the parent compound, together with a reasonable total conductivity. All these properties make this material a good cathode candidate for solid oxide fuel cells performing at intermediate temperatures (IT-SOFC).

## Figures and Tables

**Table 1:** Structural parameters for SrCo_0.95_Ru_0.05_O_3−δ_ refined from neutron powder diffraction (NPD) data at 25 °C.

Crystal Data							
a = 3.87158 (8) Å				c = 7.7928 (2) Å			
V = 116.81 (1) Å^3^				Z = 2			
**Refinement**							
R_p_ = 3.07%				R_wp_ = 3.98%			
R_exp_ = 2.59%				R_Bragg_ = 8.04%			
χ^2^ = 2.41				3199 data points			
**Fractional Atomic Coordinates and Isotropic or Equivalent Isotropic Displacement Parameters (Å^2^)**							
**Atoms**	**x**	**y**		**z**		U_iso_*/U_eq_	**Occupation (<1)**
Sr	0.50000	0.50000		0.2572 (4)		0.0103 (9)*	0.90000
Ba	0.50000	0.50000		0.2572 (4)		0.0103 (9)*	0.10000
Co1	0.00000	0.00000		0.00000		0.005 (2)*	
Co2	0.00000	0.00000		0.50000		0.020 (4)*	0.95000
Ru2	0.00000	0.00000		0.50000		0.020 (4)*	0.05000
O1	0.50000	0.00000		0.00000		0.0112 (15)*	
O2	0.00000	0.00000		0.7696 (7)		0.027 (3)	0.921 (4)
O3	0.50000	0.00000		0.50000		0.044 (4)	0.824 (2)
**Anisotropic Displacement Parameters (Å^2^)**							
**Atoms**	**U^11^**	**U^22^**	**U^33^**		**U^12^**	**U^13^**	**U^23^**
O2	0.038 (3)	0.038 (3)	0.007 (3)		0.0000	0.00000	0.00000
O3	0.024 (3)	0.073 (6)	0.036 (4)		0.00000	0.00000	0.00000

**Table 2:** Polarization resistances and power densities (with H_2_ as a fuel) of different cathodes. For the sake of comparison, values of undoped SrCoO_3−δ_ (2H phase) are also given. For the Power Densities, LSGM and SrMo_0.8_Fe_0.2_O_3−δ_ perovskite were used as electrolyte and anode material, respectively. For the 2H phase, no power density magnitude was available.

Cathode (850 °C)	Rp (Ω cm^2^)	Power Densities (mW cm^−2^)
Sr_0.9_Ba_0.1_Co_0.95_Ru_0.05_O_3_	0.048	this work
SrCo_0.90_Ru_0.1_O_3−δ_	0.110	652 [14]
SrCo_0.95_Ru_0.05_O_3−δ_	0.070	1100 [14]
SrCo_0.9_Re_0.1_O_3−δ_	0.066	660 [10]
SrCo_0.95_Re_0.05_O_3−δ_	0.080	570 [10]
SrCo_0.95_Ti _0.05_O_3−δ_	0.016	824 [13]
SrCo_0.97_V_0.03_O_3−δ_	0.025	550 [9]
SrCoO_3−δ_ (2H-phase)	0.362	- [8]
